# Evaluation of delphinidin as a storage medium for avulsed teeth

**DOI:** 10.1186/s12903-023-02713-9

**Published:** 2023-01-14

**Authors:** Ok Hyung Nam, Sang Tae Ro, Hyeon-Woo Lee, Jaeki Jeong, Yong Kwon Chae, Ko Eun Lee, Sung Chul Choi, Sang Wook Kang

**Affiliations:** 1 Department of Pediatric Dentistry, School of Dentistry, Kyung Hee University, Seoul, Korea; 2 Department of Pediatric Dentistry, Kyung Hee University College of Dentistry, Kyung Hee University Medical Center, Seoul, Korea; 3 Department of Dentistry, Graduate School, Kyung Hee University, Seoul, Korea; 4 Department of Pharmacology, School of Dentistry, Kyung Hee University, Seoul, Korea; 5 Department of Oral and Maxillofacial Pathology, School of Dentistry, Kyung Hee University, 26 Kyungheedae-Ro, Dongdaemun-Gu, Seoul, 02447 Korea

**Keywords:** Avulsed tooth, Periodontal ligament cell, Storage medium, Tooth replantation

## Abstract

**Background:**

Delphinidin (DP), an anthocyanidin found in blueberries, has antioxidant and anti-inflammatory effects. This study aimed to investigate the efficacy of DP as a storage medium for avulsed teeth.

**Methods:**

Human periodontal ligament cells were cultured and exposed to DP solution (10, 50, and 100 μM), Dulbecco’s modified Eagle’s medium, Hank’s balanced salt solution and tap water. Cell counting kit-8 assays were performed after 0.5, 1, 6, and 24 h to measure the cell viability. Nitric oxide assays and gelatin zymography were performed to evaluate the anti-inflammatory effects of DP. Reverse transcription-polymerase chain reaction was used to determine the expression levels of inflammatory cytokines.

**Results:**

The viability of periodontal ligament cells was greatest at 100 μM DP. At 1 h, 100 μM DP decreased nitric oxide synthesis (*p* < .0167). Matrix metallopeptidase-9 activity was inhibited by DP in a dose-dependent manner (*p* < .0167). Moreover, treatment with 100 μM DP decreased the expression levels of tumor necrosis factor (TNF)-α, interleukin (IL)-6, and IL-8 in periodontal ligament cells (*p* < .0167).

**Conclusions:**

Within the limits of this study, DP preserved the viability and suppressed the inflammatory response of periodontal ligament cells. These findings suggest that DP could be promising for preservation of avulsed teeth.

## Background

Tooth avulsion is a type of traumatic dental injury in which a tooth is completely displaced from its socket. To promote the healing of avulsed teeth, immediate replantation is advised [1]. In a previous clinical study, favorable healing was observed in the majority of immediate replantation cases, whereas only 18% of delayed replantation cases showed favorable healing [2]. It is crucial to preserve the viability of the periodontal ligament (PDL) cells of avulsed teeth to achieve a favorable prognosis. Until the teeth are replanted, avulsed teeth should be stored in a suitable storage medium to keep PDL cells viable. Previous studies have suggested easily available products in markets to preserve the avulsed teeth, including Gatorade (Thirst Quencher Starfruit, Gatorade, Chicago, IL), contact lens solution, Save-a-tooth (Phoenix-Lazerus Inc., Shartlesville PA, USA), and EMT Toothsaver (SmartPractice.com, Phoenix, AZ, USA) [3–5]. Several studies have also reported natural compounds such as propolis, red mulberry, green tea extract as potential storage media for avulsed teeth [6–9]. However, previous studies regarding natural compounds on this topic have mainly focused on cell viability testing rather than anti-inflammatory effects in relation to PDL cell preservation.

Delphinidin (DP) is an anthocyanidin found in blueberries and other pigment-rich vegetables. DP has anti-inflammatory and antioxidant properties [10]. Many hydroxyl groups can be found in the molecular structure of DP. The 3-dihydroxyl structure of the B-ring is responsible for the majority of its antioxidative effect [11]. According to a previous study that evaluated the antioxidative activity of anthocyanins, DP exhibited the highest radical-neutralizing potency [12]. DP exerts inhibitory effects on pro-inflammatory cytokines in tumor necrosis factor (TNF)-α-exposed human rheumatoid arthritis synovial cells by downregulating the nuclear factor (NF)-κB pathway [13]. Furthermore, in osteoarthritic chondrocytes, DP reduced the expression of cyclooxygenase-2 mRNA and the production of prostaglandin E2 induced by interleukin (IL)-1β, which is also related to external root resorption [14, 15].

DP showed favorable effects in inflammatory conditions. In this sense, we hypothesized that DP could exert anti-inflammatory, antioxidant, and cell-preserving effects on PDL cells of avulsed teeth. Therefore, this study aimed to evaluate the biological effects of DP on human PDL cells as a candidate for a storage medium for avulsed teeth. The null hypothesis was that DP treatment had no impact on inflammatory responses of PDL cells.

## Methods

### Cell culture of human PDL cells

Human periodontal ligament fibroblasts (CAT No. 2630) cells were purchased from ScienCell Research Laboratories (Young Sciences Inc. Seoul, Korea) and cultured in Dulbecco’s modified Eagle’s medium (DMEM; Gibco BRL, Life technologies, Grand Island, NY) with 10% fetal bovine serum, 100 U/mL penicillin, and 100 μg/mL streptomycin at 37 °C and 5% CO_2_. Cell culture medium was exchanged every 2 days. Cells at passage 4 were used for subsequent experiments.

### Preparation of PDL cell storage media

DP was purchased from Sigma Aldrich (Seoul, Korea) and dissolved in dimethyl sulfoxide at a stock concentration of 10 mM. DP solutions (10, 50, and 100 μM) were prepared by diluting the DP stock solution in DMEM. A pure DMEM (0 μM DP) and Hank’s balanced salt solution (HBSS; Sigma-Aldrich, Seoul, Korea) were used as positive controls. Tap water was also tested as a negative control.

### Cell viability test

The viability of PDL cells was measured using the cell counting kit-8 assay (CCK-8; Dojindo Molecular Technologies), as described previously [16]. PDL cells were seeded into a 96-well plate at 5 × 10^3^ cells/well in 200 μL of culture media. After incubation for 24 h for cell adhesion, the culture media was replaced with the prepared storage media: (a) DMEM (0 μM DP), (b) 10 μM DP, (c) 50 μM DP, (d) 100 μM DP, (e) HBSS, and (f) tap water. Cells were exposed to the medium for 30 min and 1, 6, and 24 h. Then, 20 μL of CCK-8 solution was added to the plates and incubated for 2 h at 37 °C. Cell viability was determined by measuring the optical density at 450 nm using a Benchmark Plus Multiplate Spectrophotometer (Bio-Rad, Hercules, CA, USA). Relative cell viability was compared to the differences in optical density.

### Nitric oxide (NO) assay

First, 2 × 10^5^ PDL cells were seeded into a 6-well plate and incubated at 37 °C with 5% CO_2_ for 24 h. The culture medium was removed from the wells. Based on the results of the cell viability test, 2 mL of DMEM (0 μM DP) and DP solutions at concentrations of 50 μM and 100 μM were tested. After either 1 or 6 h storage, cell supernatants were collected from the well of DMEM after 1 h storage, 50 and 100 μM DP solution after 1 h storage, DMEM after 6 h storage, and 50 and 100 μM DP solution after 6 h storage. Then, NO assay was performed using NO Plus Detection Kit (iNtRON Biotechnology, Inc., Seoul, Korea). Briefly, each 100 μL of supernatant was mixed with N1 buffer (50 μL) in 96-well plate. Next, 50 μM of N2 buffer was applied to the plate after 10 min. The absorbance of each well was measured at 540 nm. Quantification of Nitrite concentration was calculated based on a nitrite standard curve according to the manufacturer's protocol. All assays were performed in triplicate.

### Assessment of the matrix metallopeptidase (MMP)-9 activity via gelatin zymography

Gelatin zymography was performed to assess the MMP-9 activity. Same cell supernatants as NO assay were used for gelatin zymography. For each sample, the cell supernatant was mixed at a 1:4 (sample buffer:cell supernatant) ratio with non-reducing sample buffer (125 mM Tris–HCl (pH 6.8), 2% SDS, 10% glycerol, and 0.001% bromophenol blue) and electrophoresed on 10% polyacrylamide gels containing 0.3% gelatin. After electrophoresis, the gels were washed twice with 2.5% Triton X-100 for 30 min at room temperature and incubated 24 h at 37 °C in an incubation buffer containing 50 mM Tris–HCl (pH 7.5), 10 mM CaCl_2_, and 0.01% NaN_3_. The gels were stained with 0.25% Coomassie Blue R-250 and destained with 5% methanol and 8% acetic acid. Gelatinolytic bands were observed as clear zones against a blue background, and the intensity of the bands was estimated using the ImageJ software (National Institutes of Health, Bethesda, MD, USA). All zymography tests were performed in triplicate.

### RNA isolation and reverse transcription-quantitative polymerase chain reaction (RT-qPCR)

According to the manufacturer’s instructions, an AccuPrep Universal RNA Extraction Kit (K-3140, K-3141; Bioneer, Daejeon, Korea) was used to extract RNA from the same cells which had used for NO assay and gelatin zymography. 400 μL of RB buffer was added and vortexed. 300 μL of ethanol (80%) was then added and subsequently mixed with a pipette. Each sample was transferred to a binding column and centrifuged at 14,000 rpm for 20 s. After adding 700 μL of RWA 1 buffer to each tube and centrifuging at 14,000 rpm for 20 s, the remaining solution was discarded. After that, 500 μL of RWA 2 buffer was added and centrifuged at 14,000 rpm for 20 s. To remove ethanol, each column was centrifuged for 1 min. For elution, the columns were transferred to fresh 1.5 mL tubes with 50–200 μL of ER buffer and was centrifuged at 10,000 rpm for 1 min.

Next, RT-qPCR was performed. Briefly, 5 μg of total RNA was converted into complementary DNA (cDNA) using a MiniAmp Plus Thermal Cycler. Next, the QuantStudio 5 Real-Time PCR Ultimate Simplicity System was used to measure SYBR green fluorescence with a commercial reagent (Power SYBR Green Master Mix; Thermo Fisher Scientific, Carlsbad, CA, USA). The cycling conditions were as follows: denaturation for 10 min at 95 °C, followed by 40 amplification cycles of denaturation for 10 s at 95 °C and annealing for 45 s at 59 °C. cDNA levels were normalized to glyceraldehyde 3-phosphate dehydrogenase (GAPDH) levels using the 2^–ΔΔCt^ method. The primer sequences used for PCR were as follows: TNF-α, (F) 5′—CTCTTCTGCCTGCTGCACTTTG—3′, (R) 5′—ATGGGCTACAGGCTTGTCACTC—3′; IL-6, (F) 5′—AGACAGCCACTCACCTCTTCAG –3′, (R) 5′—TTCTGCCAGTGCCTCTTTGCTG—3′; IL-8, (F) 5′—GAGAGTGATTGAGAGTGGACCAC—3′, (R) 5′—CACAACCCTCTGCACCCAGTTT—3′; GAPDH, (F) 5′—GTCTCCTCTGACTTCAACAGCG—3′, (R) 5′—ACCACCCTGTTGCTGTAGCCAA—3. All experiments were performed in triplicate.

### Statistical analysis

Statistical analysis was conducted using one-way analysis of variance followed by Tukey’s HSD post-hoc test using SPSS software (Version 21.0; IBM Corp., Armonk, NY, USA). Data are expressed as the mean ± standard error of the mean. *P-*values < 0.0167 with Bonferroni corrections were considered to be statistically significant.

## Results

**Fig. 1:**
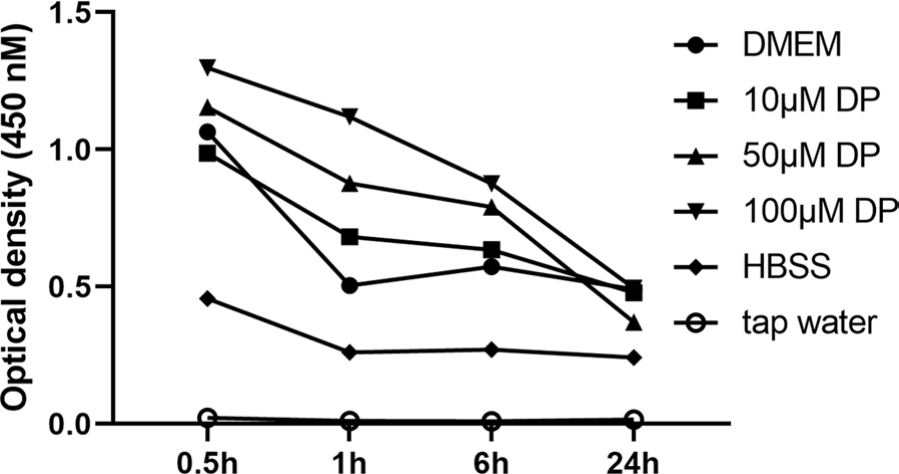
Mean optical density (450 nm), which represents the periodontal ligament (PDL) cell viability for each storage medium at different storage time points

Figure 1 shows the average absorbance values for the viability of PDL cells stored in each storage medium. At all storage time periods, DMEM and DP solutions showed higher optical densities than HBSS and tap water. In addition, at all storage time periods, 100 μM DP solution had the highest average absorbance value, followed by 50 μM DP solution at all storage time periods, except 24 h.

**Fig. 2:**
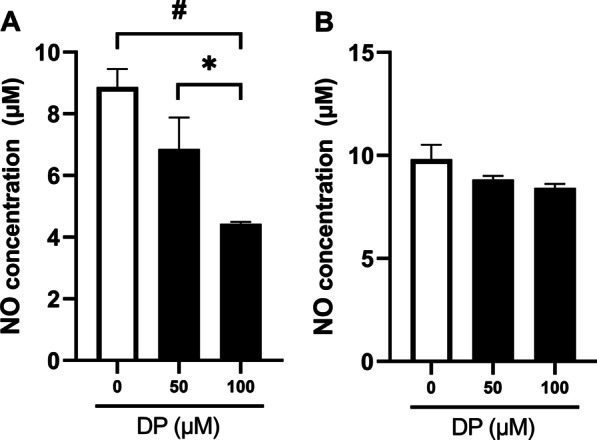
Nitric oxide (NO) production in the storage medium was analyzed using the NO detection kit. Data are presented as the mean ± standard error of the mean, *n* = 3. **A** At 1 h experiment, *p < .0167 compared with 50 and 100 μM DP solution groups, ^#^*p* < .01 compared to DMEM and 100 μM DP solution groups. **B** After 6 h, there were no significant differences

In the 1 h storage NO detection experiment, PDL cells treated with 100 μM DP solution produced significantly less NO than those treated with DMEM and 50 μM DP solution. The concentration of NO decreased as the DP concentration increased. (Fig. 2a). At 6 h storage, there was no significant difference in NO detection among the DMEM, 50 μM DP, and 100 μM DP solution-treated groups. However, when the concentration of DP was increased, the production of NO was decreased (Fig. 2b).

**Fig. 3:**
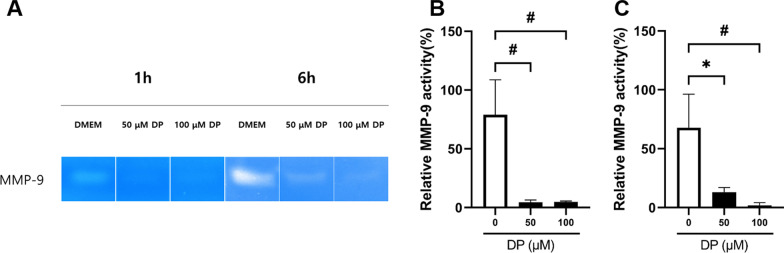
Determination of MMP-9 activity under DP treatment. Data are presented as the mean ± standard error of the mean, *n* = 3. **A** Zymography of PDL cells under DP treatment. Activity of MMP-9 in each storage media was determined by gelatin zymogram. **B** Densitometric comparsion of relative MMP-9 activity at 1 h. **C** Densitometric comparsion of relative MMP-9 activity at 6 h. *p < .0167 and ^#^*p* < .01 indicated that the difference between groups was statistically significant

Zymography demonstrated that the main gelatinase released by PDL cells migrated at 84 kDa, which is representative of active MMP-9, as shown in Fig. 3a. However, gelatinolytic bands at 72 kDa (MMP-2 pro-form), 62 kDa (MMP-2 active form), and 92 kDa (MMP-9 pro-form) were infrequently observed. These experiments showed that the suppression of MMP-9 activity was more pronounced in the 50 and 100 μM DP solution groups than in the DMEM group. In both 1 and 6 h experiments, the activity of MMP-9 was significantly inhibited by DP (Fig. 3b and c).

**Fig. 4:**
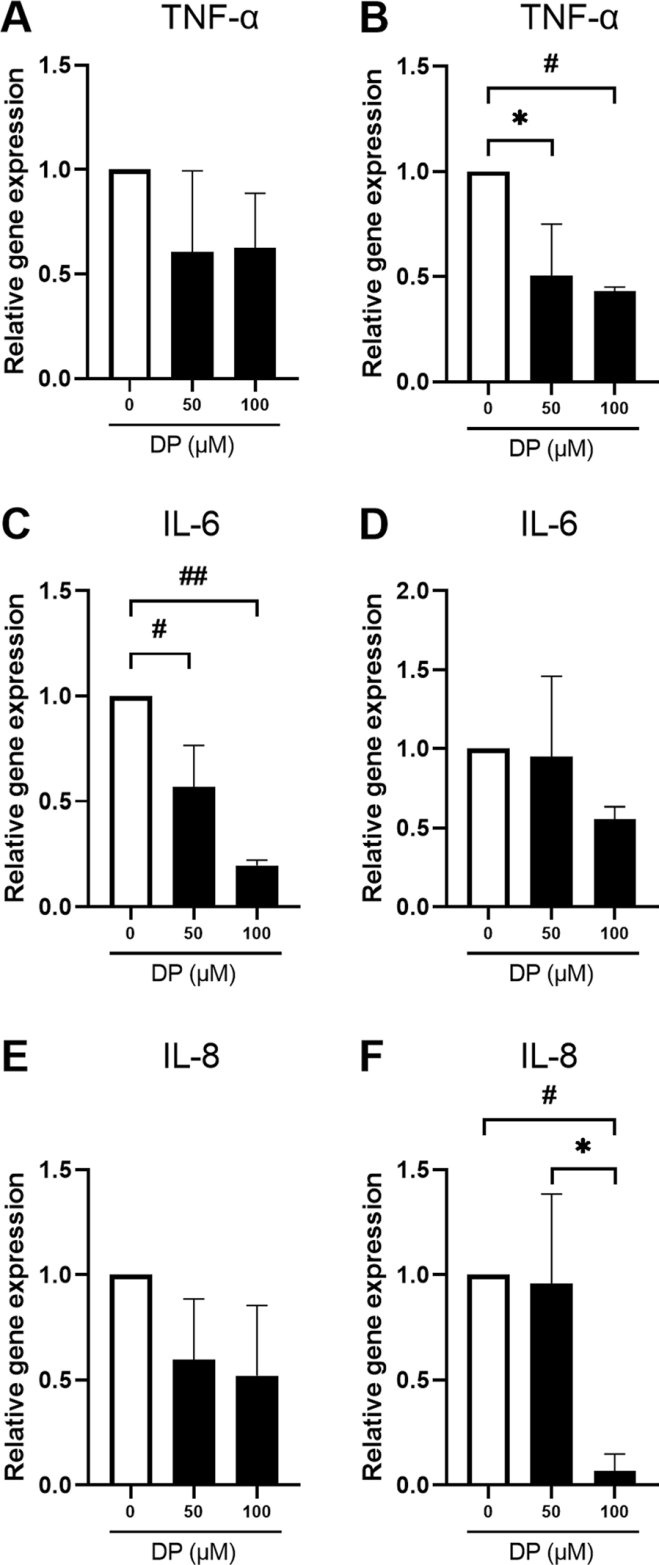
Relative expression levels of pro-inflammatory mediators. The expression levels of **A** TNF-α at 1 h **B**. TNF-α at 6 h, **C** IL-6 at 1 h, **D** IL-6 at 6 h, **E** IL-8 at 1 h, **F** IL-8 at 6 h were analyzed using RT-qPCR. Data are presented as the mean ± standard error of the mean, *n* = 3. *p < .0167, ^#^*p* < .01, ^##^*p* < .001 indicated that the difference between groups was statistically significant

At 1 h of storage, RT-qPCR analysis showed that the relative gene expression levels of IL-6 in 50 and 100 μM DP solution groups decreased significantly compared to those in the DMEM group (Fig. 4). Although there was no significant difference between the groups in terms of the expression levels of TNF-α and IL-8, the relative expression levels in 50 and 100 μM DP solution groups were downregulated compared to those in the DMEM group. RT-qPCR analysis after 6 h of storage revealed that the 50 and 100 μM DP solution groups had considerably lower relative gene expression of TNF-α than the DMEM group. The expression of IL-6 did not differ significantly across the groups. IL-8 expression was considerably decreased in the 100 μM DP solution group than in the DMEM and 50 μM DP solution groups, although 50 μM DP solution did not significantly differ from the DMEM group.

## Discussion

Our results showed that DP preserved the viability of PDL cells via anti-inflammatory effects. DP exerted anti-inflammatory effects on PDL cells by inhibiting the expression of MMP-9, TNF-α, IL-6, and IL-8.

Between immediate and delayed replantation, different levels of PDL healing of avulsed teeth are observed. These differences largely depend on the inflammatory response of PDL tissue. A previous study that evaluated the biological responses of PDL tissue following immediate and delayed replantation found a significant relationship between TNF-α signaling via the NF-κB pathway and differences in the healing responses of the periodontal tissues of replanted teeth [17]. Therefore, immediate replantation is the best option for a good prognosis in avulsed teeth [18]. However, replanting an avulsed tooth is frequently postponed because of insufficient knowledge or confidence in the replantation procedure [19, 20]. If the replantation of an avulsed tooth is delayed, root resorption in such tooth may occur [21].

To ensure the viability of PDL cells when the replantation of the avulsed tooth is delayed, the tooth should be maintained in an appropriate storage medium [22, 23]. Tooth storage media for avulsed teeth must be capable of retaining cell viability, have physiological pH and osmolality similar to that in the oral environment, provide nutrition and growth factors for cell metabolism, and be readily available and accessible [24, 25]. Storing the avulsed tooth in an antioxidant-rich storage medium may help to lower the rates of root resorption following replantation [26]. Addition of anti-inflammatory characteristics to storage media may aid in preventing root resorption and improving the results of replanted teeth. Thus, we selected DP, which exerts antioxidant and anti-inflammatory effects, for subsequent experiments.

Previous studies have demonstrated that 100 μM DP has anti-inflammatory and cell-preserving effects in human cells [27, 28]. In this study, 100 μM DP showed the highest PDL cell viability, which was consistent with the studies. To determine whether DP exerts anti-inflammatory effects on PDL cells, NO generation by DP was tested, and a significant reduction in NO production was detected.

RT-qPCR and zymography were performed to further study the anti-inflammatory effects of DP on particular inflammatory mediators. Based on zymography data, DP considerably reduced the MMP-9 activity in a dose-dependent manner. Increased MMP-9 activity is associated with inflammatory processes, especially when the destruction of periodontal tissue is severe [29]. Bone resorption is directly correlated with MMP-9 production of the osteoclasts [30]. Ahn et al. [19] demonstrated that extra-oral time affected the expression levels of inflammatory cytokines and MMPs in replanted rat teeth. Additionally, there was a positive correlation between MMP-9 expression and longer extra-oral periods in rat teeth that had been replanted. These results suggest that DP can reduce MMP-9 expression, which in turn can reduce the inflammatory response of avulsed teeth following delayed replantation. Along with MMP-9, MMP-2 is a well-known gelatinase that is involved several inflammatory processes, collagen remodeling, and alveolar bone regeneration [31]. MMP-2 expression, however, was not detected in the zymography used in this study. This indicates that in contrast to the in vitro avulsion model, MMP-2 may be more engaged in bone turnover or remodeling of the extracellular matrix [32].

In this study, RT-qPCR analysis showed that DP significantly reduced the expression levels of pro-inflammatory cytokines, such as TNF-α, IL-6, and IL-8. These results suggest that DP exerts anti-inflammatory effects. In our study, DP significantly inhibited TNF-α expression. Previous studies using DP support this finding [14, 33]. Another study found that delphinidin-3-O-glucoside prevents the interaction of TNF-proteins by directly binding to the TNF-receptor [34]. TNF-α is a key pro-inflammatory mediator in periodontal tissue destruction [35]. Other pro-inflammatory mediators, such as IL-1β, IL-6, IL-8, and MMPs, are produced as a result of TNF-α production [33]. TNF-α significantly reduces the PDL cell migration and inhibits PDL regeneration [36]. A previous RNA-seq study demonstrated that PDL cells stored for a long time, even when stored in HBSS or milk, could experience an inflammatory response [16]. This RNA-seq study further showed that the TNF-α signaling pathway was significantly remarked in this response.

Additionally, IL-6 and IL-8 play significant roles in the development of inflammatory responses, osteoclast production, and alveolar bone resorption [37]. IL-1β, IL-6, and TNF-α induce classical hallmarks of inflammation, and they cooperate with one another in their actions [38]. In this study, DP significantly inhibited the expression of IL-6 and IL-8, which was consistent with previous studies. In a previous study, rats with spinal cord injuries received (DP) therapy, which markedly decreased their levels of TNF-α and IL-6 activity [39]. In another study on the effects of topical DP treatment on inflammatory skin disease, DP treatment reduced lesion size and mRNA expression levels of the inflammatory cytokines, TNF-α, IL-6, and IL-8 [40]. Similarly, DP treatment reduced IL-6, IL-8, and TNF-α production in a 3D human skin model [41].

However, our study has some limitations. First, the clinical scenarios of avulsed teeth were not completely simulated. Further studies are needed to determine the effect of DP on PDL cells that have been bench-dried prior to DP treatment. Next, our study only evaluated the effect of DP at the cellular level. To further clarify the efficacy of DP as a storage medium for avulsed teeth, animal and human studies will be conducted in the future.

## Conclusions

In conclusion, this study demonstrates that DP may exert therapeutic effects on PDL tissues of avulsed teeth before delayed replantation. This suggests that DP can maintain the survival of PDL cells and prevent inflammatory reactions in delayed replanted teeth. Future studies using animal models are needed to evaluate the potential of DP in vivo.

## Data Availability

All data generated or analyzed from this study are included in this published article.

## Abbreviations

PDL: Periodontal ligament
DP: Delphinidin
TNF-α: Tumor necrosis factor-α
IL-1β: Interleukin-1β
DMEM: Dulbecco’s modified Eagle’s medium
HBSS: Hank’s balanced salt solution
CCK-8: Cell counting kit-8
NO: Nitric oxide
MMP-9: Matrix metallopeptidase-9
RT-qPCR: Reverse transcription-quantitative polymerase chain reaction
cDNA: Complementary DNA
